# Host and Symbiont Jointly Control Gut Microbiota during Complete Metamorphosis

**DOI:** 10.1371/journal.ppat.1005246

**Published:** 2015-11-06

**Authors:** Paul R. Johnston, Jens Rolff

**Affiliations:** 1 Freie Universität Berlin, Berlin, Germany; 2 Berlin-Brandenburg Institute of Advanced Biodiversity Research (BBIB), Berlin, Germany; Stanford University, UNITED STATES

## Abstract

Holometabolous insects undergo a radical anatomical re-organisation during metamorphosis. This poses a developmental challenge: the host must replace the larval gut but at the same time retain symbiotic gut microbes and avoid infection by opportunistic pathogens. By manipulating host immunity and bacterial competitive ability, we study how the host *Galleria mellonella* and the symbiotic bacterium *Enterococcus mundtii* interact to manage the composition of the microbiota during metamorphosis. Disenabling one or both symbiotic partners alters the composition of the gut microbiota, which incurs fitness costs: adult hosts with a gut microbiota dominated by pathogens such as *Serratia* and *Staphylococcus* die early. Our results reveal an interaction that guarantees the safe passage of the symbiont through metamorphosis and benefits the resulting adult host. Host-symbiont “conspiracies” as described here are almost certainly widespread in holometobolous insects including many disease vectors.

## Author Summary

The majority of animals are holometabolous insects and change dramatically through development. They undergo a dramatic transformation from a larval stage, adapted to feed, to an adult separated by a pupal stage. During this pupal stage the majority of the organs are renewed including the gut. This creates a risky situation that we study here: when the gut is renewed insects risk losing beneficial microbiota while simultaneously being at risk of opportunistic infection. Here, by manipulating host and symbiont we show how host and symbiont succeed in jointly controlling opportunistic pathogens. If one or both of the partners are compromised, opportunistic pathogens dominate the gut microbiota resulting in increased mortality. These findings may be broadly applicable to insects with complete metamorphosis, including many disease vectors.

## Introduction

The vast majority of animal species are insects [1,2]. Most species of insects are holometabolous, including important vectors of infectious disesases (sandflies, mosquitoes) and model organisms (*Drosophila melanogaster*, *Galleria mellonella*) with distinct larval and adult stages separated by metamorphosis, which entails dramatic remodeling of external and internal anatomy [3,4]. The evolutionary advantage of metamorphosis is usually explained by the adaptive decoupling hypothesis [5]: traits in larvae and adults are genetically decoupled, facilitating adaptation to life-stage specific selection [6]. Anatomical re-organization of the body, however, poses a significant problem during the replacement of the gut, as the gut hosts a microbiota. Either the organism must eradicate and subsequently re-establish the microbiota from the environment, or it must maintain its microbiota while preventing opportunistic microbes from entering the hemocoel and causing infections.

Early studies clearly demonstrated the presence and maintenance of bacteria in the gut during metamorphosis in Lepidoptera and Diptera [7–9], and more recent work has described the same phenomenon in Coleoptera [10], Diptera [11], Lepidoptera [12], and Hymenoptera [13]. Two competing mechanisms have been proposed to explain the composition of the retained gut microbiota. One explanation holds that bacterial competition drives the composition of the adult gut microbiota [14,15]. Alternatively, the host immune system plays an important role in shaping the gut microbiota [16,17]. However despite continued interest in insect gut immunity [18], the interaction between host immunity and bacterial competition during complete metamorphosis has remained unstudied.

Here we exploit an ancient [19], facultative and prevalent [20–26] symbiosis between Lepidoptera and enterococci to reconcile these approaches by studying the role of host immunity and bacterial competition during metamorphosis within a single system. The gut microbiota of Lepidoptera is limited to a handful of bacterial species that varies with habitat and diet but often is dominated by enterococci that persist through metamorphosis [12]. In the lepidopteran gut, enterococci interact with pathogens through (i) competitive exclusion (ii) attenuation by direct antagonism or (iii) eliciting protective host immune responses and provide lepidopterans including *Galleria mellonella* with protection against one of the most virulent entomopathogens, *Bacillus thuringiensis* [21,27–29] (reviewed in [30]).

In Lepidoptera, the contents of the gut lumen and the peritrophic matrix are purged at the onset of metamorphosis. Basal midgut stem cells proliferate and differentiate to form a continuous layer beneath the larval gut epithelium where lysozyme accumulates in apical vacuoles [16]. Following ecdysis of the larval epithelium, the vacuole contents are released into the gut lumen producing a burst of antibacterial activity that is presumed to prevent septicemic infection [16]. The detached larval epithelium undergoes autophagy and apoptosis and degenerates as the 'yellow body' [31]. Bacteria that resist mechanical and immunological exclusion by the host during pupation, compete intensely for colonization of the pupal gut as has been demonstrated in flies [14].

In the model lepidopteran *Galleria mellonella*, *Enterococcus mundtii* (syn. *Streptococcus faecalis* Andrewes and Horder) is passed from female to offspring via the surface of the egg [25]. *In vitro* observations of lepidopteran gut microbes imply that *E*. *mundtii* has the highest abundance, in adults it is often the only detectable microbe in the gut, and that this is mediated by synergy between lysozyme and a broad-spectrum bacteriocin [28]. As is common in many Lepidoptera [32], *G*. *mellonella* adults lack functional mouthparts and therefore additional microbes cannot be acquired during adult life.

Using the *Galleria*-*Enterococcus* symbiosis we tested the hypothesis that host and symbiont interact to determine the adult gut microbiota. We manipulated host gut immunity and bacterial competitive ability during metamorphosis in a full factorial fashion. Based on these findings we studied fitness consequences of altered microbiotas for adult hosts.

## Results and Discussion

Previous studies show that lysozyme is important in host-microbe interactions in the pre-pupae of another lepidopteran [16], and in *G*. *mellonella* a synergistic interaction between C-type lysozyme and antimicrobial peptides was recently demonstrated [33]. Based on the expression of a C-type lysozyme (Swiss-Prot accession P82174) in the gut during metamorphosis (S1 Fig) and the reported synergism of *G*. *mellonella* lysozyme we hypothesized that lysozyme mediates changes in the microbiota during metamorphosis. We knocked down lysozyme expression using RNAi in insect hosts that were colonized by either *E*. *mundtii* strain G2-mun+, which produces the broad-spectrum bacteriocin mundticin [34], or by *E*. *mundtii* strain G2-mun-, which is unable to express the mundticin-encoding gene *munA*.

To test the impact of these manipulations we cured the gut microbiota from final-instar larvae using antibiotics and re-inoculated these individuals with either *E*. *mundtii* G2-mun+ or G2-mun-, and reared them on conventional non-sterile diet until pupation.

Using a combination of 16S rRNA gene amplicon sequencing, qPCR, and conventional bacterial culturing we monitored the composition of the gut microbiota during the larval-pupal molt as well as after adult eclosion.

**Fig 1:**
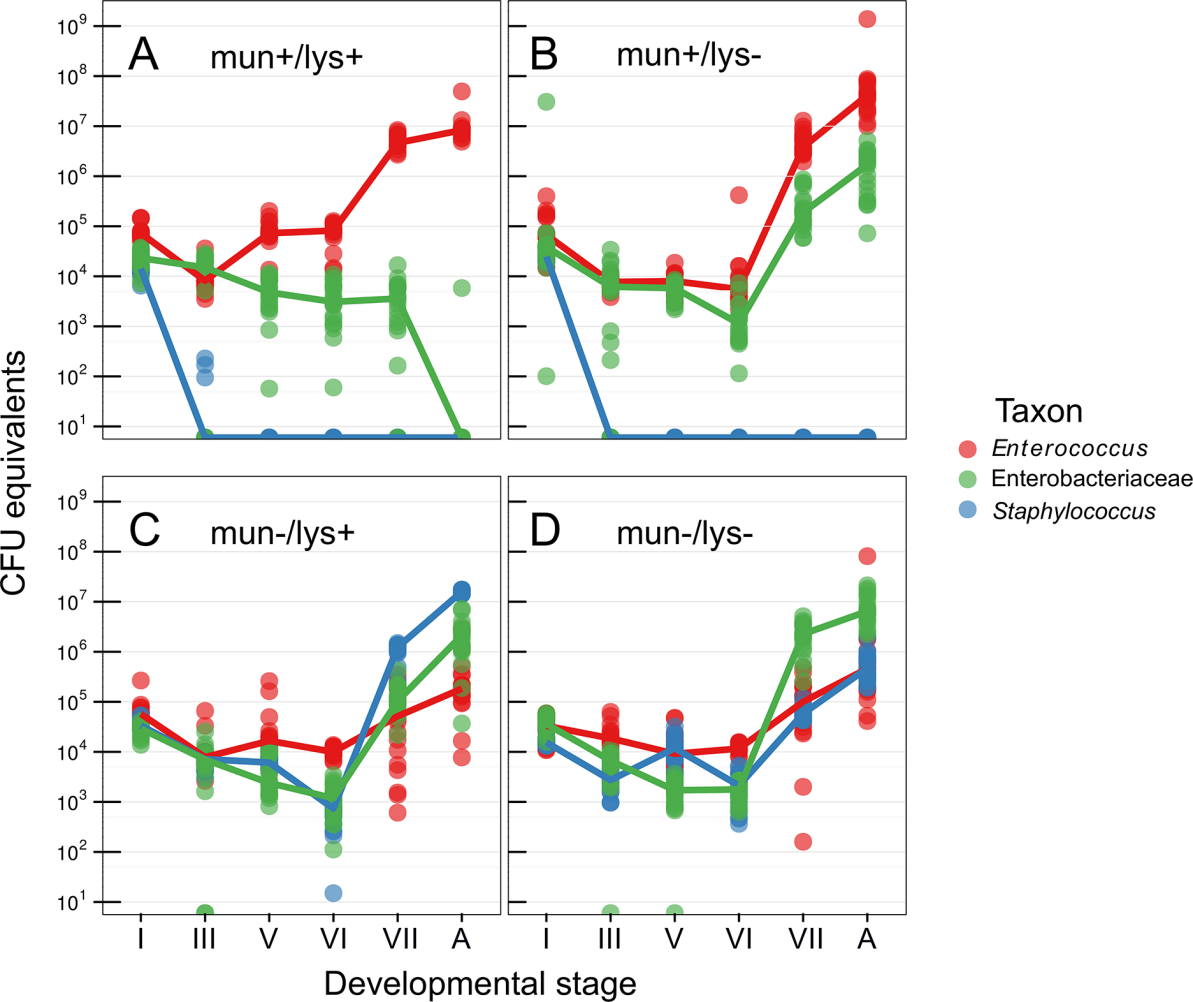
Abundance of bacteria during host metamorphosis. Using 16S rRNA gene qPCR the microbiota was sampled across metamorphosis in (A) wild-type hosts with bacteriocin-producing *Entercoccus mundtii* G2 symbiont, (B) immunocompromised hosts where RNAi was used to knock down lysozyme gene expression, (C) wild-type hosts with mutant *Entercoccus mundtii* symbionts that do not produce bacteriocin, and (D) both immunocompromised hosts and mutant symbionts. Roman numerals correspond to precise stages of the larval-pupal molt (see materials and methods). A, adult.

The microbiota of the wild-type host during pupation was increasingly dominated by *Enterococcus*, whereas *Serratia* and *Staphylococcus* were undetectable in the adult stage by culturing, 16S rRNA gene amplicon sequencing (S2 Fig, S1 Table), and 16S rRNA gene qPCR (Fig 1). Host lysozyme-knockdown resulted in a significant increase in Enterobacteriaceae and persistence into the adult stage (T = -25.145, df = 456, *p* = <0.0001), which appear to be entirely composed of a *Serratia sp*. When the host is instead associated with *E*. *mundtii* G2-mun- (which does not produce the bacteriocin munditicin), *Staphylococcus* becomes highly abundant after pupation (T = -96.48, df = 456, *p* <0.0001, Fig 1) and *Enterococcus* are reduced by two orders of magnitude. When both host and symbiont are disenabled, Enterobacteriaceae (*Serratia sp*.) dominates (T = -28.655, df = 456, *p* < 0.0001) and again *Enterococcus* are strongly reduced on reaching the adult stage (T = 10.290, df = 448, *p* < 0.0001, Fig 1).

**Fig 2:**
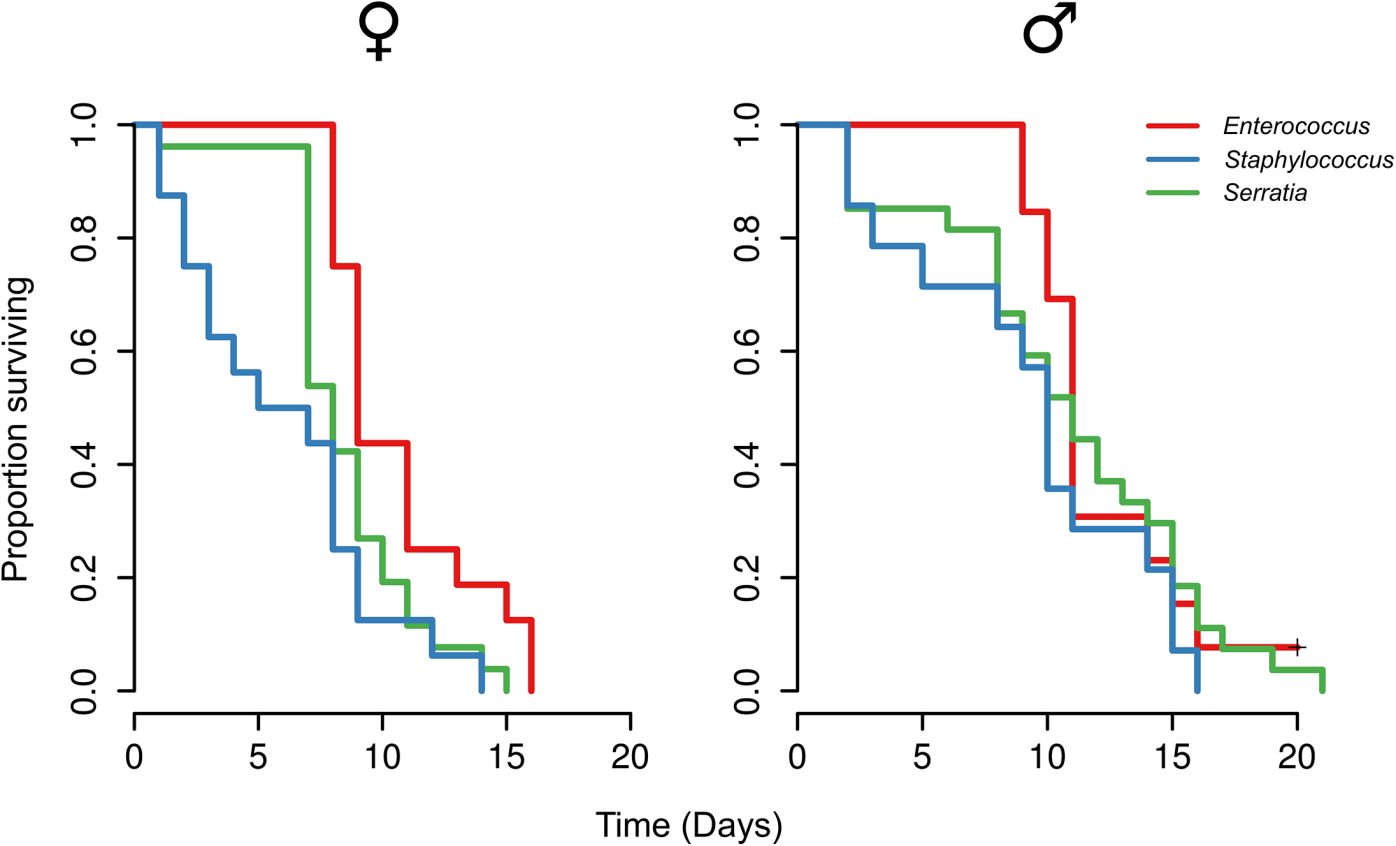
Survival of adults with experimentally defined gut microbiota. Larvae were re-inoculated with a defined microbiota comprising either wild-type *Enterococcus mundtii*, *Staphylococcus* sp. or *Serratia* sp. based on previous results (Fig 1).

We found that the gut microbiota composition significantly changes when either the symbiont and/or host and symbiont were both disenabled (Fig 1). Based on these results we therefore inoculated mature larvae, that were first cleared of their microbiota using antibiotics (‘re-inoculated larvae’), with a defined microbiota comprising either wild-type *Enterococcus mundtii*, *Staphylococcus sp*. (reflecting the results when the symbiont is disenabled) or *Serratia sp*. (as found when both host and symbiont are disenabled), which were isolated from *G*. *mellonella* larvae. This made it possible to investigate fitness costs of a defined microbiota without the confounding effects of the manipulation of the host immune system by RNAi or changes to symbiont competitive ability by manipulating mundticin expression. Survival of the resulting adults was monitored after eclosion. Independent of host sex, *G*. *mellonella* individuals with *E*. *mundtii* survived significantly longer than those with a *Staphylococcus*- (Z = -4.72, *p* = <0.0001), or *Serratia*- (Z = -2.97, *p* = 0.003) dominated microbiota (Fig 2, S2 Table). There was no difference in survival between *G*. *mellonella* adults derived from larvae that were either cured of their microbiota and maintained axenically, or which were re-inoculated with *E*. *mundtii* (S3 Fig). This supports the conclusion that the main benefit of E. mundtii mutualism is the interaction with other members of the gut microbiota.

Our study shows that host and symbiont interact to maintain a ‘healthy’ gut microbiota through complete metamorphosis. Given the protection that *E*. *mundtii* confers to the lepidopteran host [28,30] the selective advantage for the host is clear. The transmission of the gut microbiota between individuals is usually considered as mixed-mode transmission, combining vertical and horizontal transmission [35]. For the bacterial symbiont, passage through metamorphosis constitutes an important component of vertical transmission. Complete sterilization of the gut by the host would spell disaster for the symbiont. The transition of the symbiont through metamorphosis is crucial to guarantee vertical transmission to the host offspring. In social species, this can be overcome through exposure to feces of nest mates [36]. But most holometabolous insects are solitary and therefore this mechanism of re-acquisition of symbionts from conspecifics after metamorphosis is impractical. Some species of ants and weevils have resolved the transition of the microbiota through metamorphosis with bacteriocytes, crypts, and other specialized structures that harbour symbionts throughout the life cycle [37,38], this is not the case in Lepidoptera.

Our study also sheds light on an important aspect of the evolution of complete metamorphosis, a key innovation of holometabolous insects [39] that resulted in their extraordinary diversity. An understanding of the benefits and costs of complete metamorphosis is essential to explain the evolutionary success of holometabolous insects. Ecological and evolutionary models of metamorphosis are sufficient to explain the evolution of complex life cycles [5], but not of the pupal stage that defines complete metamorphosis. One adaptive explanation that has been proposed, but barely tested, is the decoupling of growth and differentiation into consecutive stages of the larva and the pupa [40]. The evolution of such a novel complex trait as the pupal stage also creates significant problems. The pupa is a sessile and hence vulnerable stage in the insect life cycle. Some parasitoids specialize on pupal hosts and display adaptations that exploit host endocrinology during metamorphosis [41]. Here, by contrast, we have identified and described a ubiquitous problem encountered by most holometabolous insect during ontogeny: the passage of symbiontic bacteria through pupation. Our data show how the host and symbiont interact to achieve this passage. Moreover, the data also suggest that the risk of opportunistic infection during the destruction of the larval gut is countered by the host through up-regulation of immune function. The destruction of the gut, and hence the abundance of danger signals, in combination with microbe-derived immune elicitors result in a strong immune response [42] as found here.

## Materials and Methods

### Bacterial strains and growth conditions

*Enterococcus mundtii* G2 was isolated from a long-term laboratory population of *Galleria mellonella* where insects were reared on a natural diet of honeycomb [43]. To cure *E*. *mundtii* G2 of its munditicin-encoding plasmid, a single colony was picked and grown in BHI broth at 42°C for 5 24-h serial passages. The resultant strain was transformed by the method of Dunny et al. [44] with either pRK1 or pRK62 which both contain the entire *mun* locus with and without a *munA* promoter, respectively [34]. Both strains express the mundticin ABC transporter protein and mundticin immunity protein (MunB and MunC respectively) [34], and enterococci are intrinsically resistant to lysozyme [45]. The resultant strains are referred to as mun+ and mun-. The wild-type *mun* locus comprises *munA*, which encodes the bacteriocin mundticin; *munB* encoding a mundticin-translocating ABC transporter; and *munC* encoding a mundticin immunity protein. *munBC* expression is under independent transcriptional control from *munA* and is driven by a promoter located between *munA* and *munB* and downstream of the *munA* terminator [34]. To enumerate gut bacteria, insects were dissected and their guts were homogenized with 5-mm steel bead using a TissueLyser (Qiagen) at 20 Hz for 10 s. Homogenates were serially diluted in sterile saline and plated onto 1/10 strength TSA (Oxoid).

### Amplification and sequencing of 16S rRNA genes

Bacterial colonies were categorized by morphotype and representatives were subjected to colony PCR with universal primers 27F and 1492r. Sanger sequencing of PCR products was performed by MWG Biotech (Ebersberg, Germany) or GATC (Konstanz, Germany). For high-throughput amplicon sequencing, total DNA was recovered from gut homogenates by bead milling and CTAB extraction [46] and 24 pools of DNA were constructed representing each combination of treatment and developmental stage using 100 ng of purified DNA from each individual. Pools were subjected to PCR with barcoded versions of the universal primers 27f and 519r. Roche multiplex identifiers were incorporated between the sequences of adaptor A and 519r to give the structure: 5'-Adaptor_A-sequencing_key-multiplex_identifier-519r-3'. PCR consisted of an initial denaturation step of 2 min at 94°C and 25 cycles of of 30 s at 94°C, 20 s at 52°C, and 60 s at 65°C. PCR products were checked by gel electrophoresis, purified with AMPure beads, and sequenced on a 454 titanium GS FLX at 24-plex per quarter pico-titer plate. Amplicon sequence data were processed using QIIME version 1.6 [47]. Sequences were assigned to operational taxonomic units according to a 97% identity threshold using uclust [48]. Data were deposited in the NCBI SRA under accession PRJNA268795.

### 16S rRNA gene qPCR

Based on the high-throughput 16S rRNA gene amplicon sequencing, taxon-specific 16S rRNA gene primers were used to quantify the three dominant taxa for the genera *Enterococcus* [49], *Staphylococcus* [50], and the family Enterobacteriaceae [49] in each individual. Dilution plating and high-throughput 16S rRNA amplicon sequencing showed the presence of three bacterial genera: *Enterococcus*, *Staphylococcus*, and *Serratia* however since *Serratia*-specific 16S rRNA gene primers could not be designed, family-specific Enterobacteriaceae primers were used to generate individual-based qPCR measurements. Standard curves were prepared using samples derived from axenic guts spiked with known quantities of either *Enterococcus mundtii* G2, *Staphylococcus sp*, or *Serratia sp*. CFU. qPCR was performed on an ABI StepOne using KAPA SYBR FAST ABI Prism mastermix (Peqlab). The resulting data were log transformed and analysed using linear models in R 3.1.3.

### Insect rearing

*G*. *mellonella* larvae were reared in the dark at 30°C on a grain-honey diet described previously [29]. Hoffman's tobacco hornworm diet was used to manipulate the *G*. *mellonella* gut microbiota. Sterile artificial diet was produced using an autoclave and heat-labile components such as Vanderzants vitamin mixture (Sigma-Aldrich) and antibiotics were dissolved in water and filter-sterilized before combing with molten diet at 60°C. To remove the gut microbiota, diet was amended with 100 μg ml^-1^ streptomycin and tetracycline (Sigma-Aldrich). Mature final-instar larvae were starved for 4 h before being transferred to sterile antibiotic-amended diet for 24 h. Removal of the microbiota was confirmed by dissecting and plating the guts of 30 randomly-chosen larvae onto TSA plates.

To associate larvae with a specific bacterial strain, sterile diet (without antibiotics) was amended with an aliquot of an overnight culture to a final concentration of 10^3^ CFU ml^-1^. Larvae were removed from sterile antibiotic-amended diet, starved for 4 h, and transferred to bacteria-amended diet for 16 h. As is common in many Lepidoptera [32], *G*. *mellonella* adults do not possess functional mouthparts therefore oral infection of adults is not possible. Larvae were subsequently starved for 4h before being returned to a conventional grain-honey diet.

### Plasmid segregational stability

The segregational stability of the plasmids pRK1 and pRK62 in *E*. *mundtii* G2 was determined according to Simon and Chopin [51] in both non-selective MRS broth (Oxoid) as well as in insect hosts. To quantify stability in broth, an overnight culture was diluted in non-selective MRS broth, grown to late exponential phase and plated onto non-selective MRS agar. 384 colonies were arrayed in duplicate onto erythromycin-selective and non-selective MRS agar. To quantify stability in insects, mature larvae were mono-associated as described above with *E*. *mundtii* G2 carrying either pRK1 or pRK62, and returned to conventional grain-honey diet to complete larval and pupal development. The frequency of vector loss was < 2.8 x 10^−3^ both in broth culture and insect hosts. In the case of broth culture, this stability is comparable to the parent vector pIL253 [51]. Upon eclosion, 10 adults carrying either mun+ or mun- strains were dissected and their guts were plated onto non-selective MRS agar (S4 Fig). 384 colonies were tested for erythromycin-sensitivity as described above. 46 randomly selected Em^R^ colonies from each larva were screened for the presence of the plasmid by colony PCR using T7 promoter-specific primers.

### Determination of the stages during metamorphosis

Complete detachment of the larval gut epithelium occurs prior to ecdysis of the larval cuticle. Therefore the staging system of Kühn and Piepho [52], as adapted by Uwo et al. [31], was used to specify the stages of midgut metamorphosis in larvae and pupae (see Uwo et al. [31] and Ellis et al. [53] for illustration). Stage I is a wandering larva that has ceased feeding and started spinning. Stemmatal pigments have not started to migrate and the midgut is empty. Stage II, is the spinning larva and pigments have started to migrate from the stemmata. Stage III, a l ater spinning larva, where the pigments have left the stemmata but are still in contact with the cuticle. The larval gut epithelium has completely detached and floats freely in the lumen. Stage IV defines a mature spinning larva and the pigments have sunk beneath the cuticle but are still visible. Stage V is the prepupa that has ceased spinning and stemmatal pigments are not visible. The midgut is laterally flattened and the detached larval gut has formed the yellow body which undergoes apoptosis. The new pupa is classed as stage VI; the cuticle has not sclerotized and is completely white. Stage VII describes a sclerotized pupa approximately 24 h after the larval-pupal molt. The midgut is cylindrical and surrounded by an extra-epithelial layer.

The migration of stemmatal pigments was monitored under a stereo microscope.

### RNAi

An internal region of the cDNA sequence encoding a C-type lysozyme, previously designated lysozyme GALME (Swiss-Prot accession P82174) [54], was amplified with T7-tailed primers Gm_Lys_T7_F1 (5'-TAATACGACTCACTATAGGGAGAGCAAGCCGAATAAAAATGGA-3') and Gm_Lys_T7_R1 (5'-TAATACGACTCACTATAGGGAGATATCTGGCAGCGGCTTATTT-3') and used as template to synthesize dsRNA using a MEGAscript T7 Kit (Ambion). In order to knockdown lysozyme GALME expression, 500 ng of purified dsRNA was injected into the hemocoel of mature final-instar larvae. RNAi efficacy was monitored throughout the larval-pupal molt by performing relative qPCR on cDNA derived from dissected guts using the primers Gm_Lys_qPCR_F1 (5'-ACTTTTACGAGATGCGGACTG-3') and Gm_Lys_qPCR_R1 (5'-TCTCATTCTCAACAAGGCACAC-3'), which target a region upstream of the region chosen as template for dsRNA synthesis, as well as S7e_forward and S7e_reverse which target the gene encoding ribosomal protein S7e [55], which was analyzed as a reference. Relative expression was calculated using the relative Ct method. cDNA was synthesized using a cDNA-Synthesis Kit H Plus (Peqlab) from 100 ng of total RNA from a pool of RNA from 3 individual insects. qPCR was performed using a peqGOLD Hot Start-Mix kit (Peqlab) and a StepOne real-time thermocycler (Applied Biosystems) according to the manufacturer’s instructions.

### Survival analysis

Mature pupae were weighed and segregated individually in plastic cups covered with muslin cloth at 30°C. Newly eclosed adults were sexed according to the forewing margin [53] and survival was recorded every 24 h. The data were analysed in R with an accelerated failure time model using the survival package [56]. The Bayesian information criterion was used to select the final model.

## Supporting Information

S1 Fig C-type lysozyme gene expression during metamorphosis. Boxplots depict three biological replicates of pools of 8–10 individual insects. Wild-type (wt), grey; RNAi, white.
(TIFF)

S2 Fig Relative abundance of bacteria during host metamorphosis. Using 16S rRNA gene amplicon sequencing the microbiota was sampled across metamorphosis in (A) wild-type hosts with bacteriocin-producing *Entercoccus mundtii* G2 symbiont, (B) immunocompromised hosts where RNAi was used to knock down lysozyme gene expression, (C) wild-type hosts with mutant *Entercoccus mundtii* symbionts that do not produce bacteriocin, and (D) both immunocompromised hosts and mutant symbionts. Roman numerals correspond to precise stages of the larval-pupal molt. A, adult; OTU, operational taxonomic unit. See S1 Table for full data.
(TIFF)

S3 Fig Survival of male and female Galleria mellonella. Adult individuals were derived from final-instar larvae which were cured of their microbiota using antibiotics. One group was re-inoculated with *E*. *mundtii* whereas the other remained axenic. No difference in survival was detected between the two groups (Accelerated failure time model, n = 106, ANOVA, DF: 1,102, p = 0.3457356).
(TIFF)

S4 Fig Densities of bacteria in *Galleria mellonella* adults. A, densities achieved by *Enterococcus mundtii* G2 with (mun plus) and without (mun minus) mundticin expression. Mundticin expression had no effect on CFU (negative binomial GLM, DF: 18, p = 0.4355). B, densities of different types of bacteria of newly-eclosed adults sampled from the survival experiment depicted in Fig 2.
(TIFF)

S1 Table OTU table. (CSV)

S2 Table Summary of accelerated failure time model for survival data shown in Fig 2. (PDF)
