# Supplementary figures and images for: Host and Symbiont Jointly Control Gut Microbiota during Complete Metamorphosis
Source: PLoS Pathog. 2015 Nov 6;11(11):e1005246. doi: 10.1371/journal.ppat.1005246 (PMC4636265; doi:10.1371/journal.ppat.1005246)

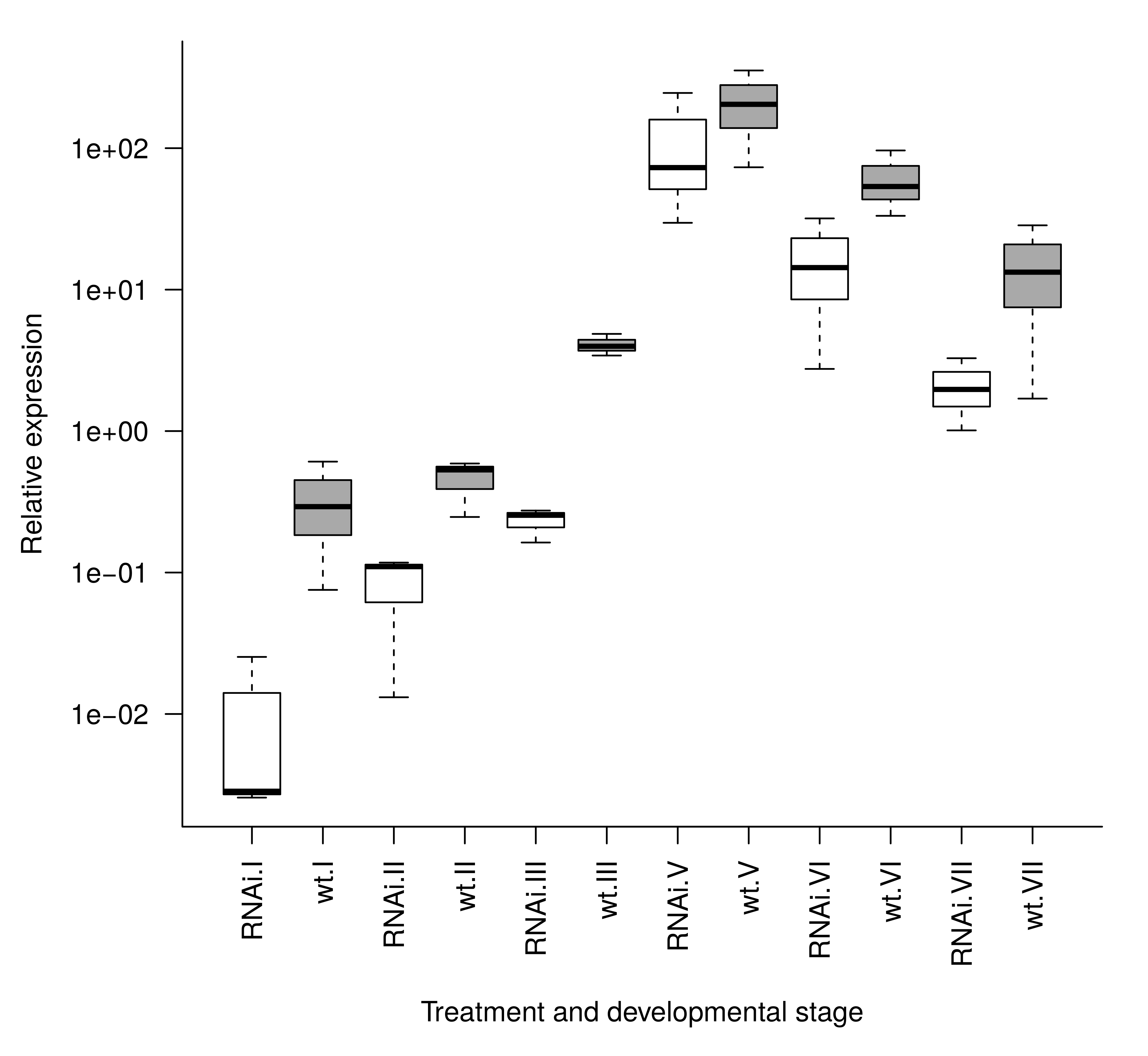

Supplement: S1 Fig — Boxplots depict three biological replicates of pools of 8–10 individual insects. Wild-type (wt), grey; RNAi, white. (TIFF) [file ppat.1005246.s001.tiff]

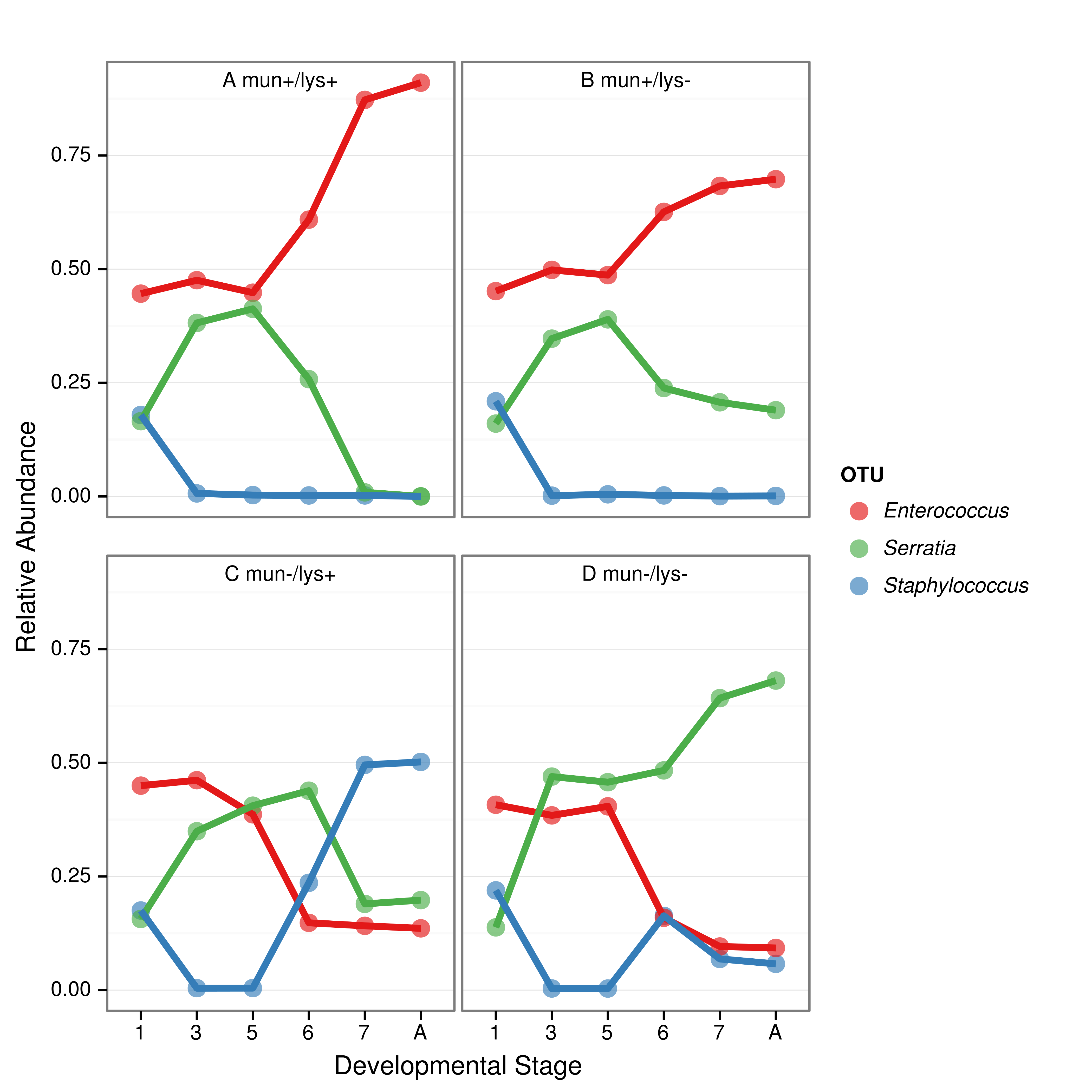

Supplement: S2 Fig — Using 16S rRNA gene amplicon sequencing the microbiota was sampled across metamorphosis in (A) wild-type hosts with bacteriocin-producing Entercoccus mundtii G2 symbiont, (B) immunocompromised hosts where RNAi was used to knock down lysozyme gene expression, (C) wild-type hosts with mutant Entercoccus mundtii symbionts that do not produce bacteriocin, and (D) both immunocompromised hosts and mutant symbionts. Roman numerals correspond to precise stages of the larval-pupal molt. A, adult; OTU, operational taxonomic unit. See S1 Table for full data. (TIFF) [file ppat.1005246.s002.tiff]

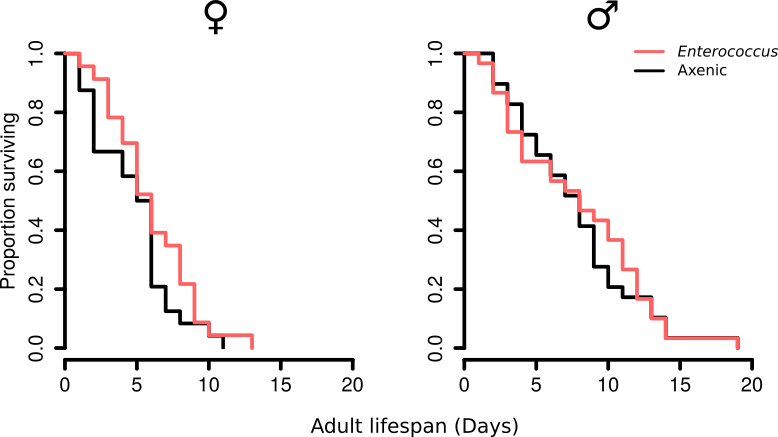

Supplement: S3 Fig — Adult individuals were derived from final-instar larvae which were cured of their microbiota using antibiotics. One group was re-inoculated with E. mundtii whereas the other remained axenic. No difference in survival was detected between the two groups (Accelerated failure time model, n = 106, ANOVA, DF: 1,102, p = 0.3457356). (TIFF) [file ppat.1005246.s003.tiff]
