# Supplementary material for: Host and Symbiont Jointly Control Gut Microbiota during Complete Metamorphosis
Source: PLoS Pathog. 2015 Nov 6;11(11):e1005246. doi: 10.1371/journal.ppat.1005246 (PMC4636265; doi:10.1371/journal.ppat.1005246)
Supplement: S2 Table — (PDF) [file ppat.1005246.s006.pdf]

**Table S2.** Summary of accelerated failure time model for survival data shown in Figure 2 .

| | Value | Std. Error | Z | p |
|---|---|---|---|---|
| (Intercept) | 15.77 | 2.1327 | 7.39 | 1.42e-13 |
| Pupal weight | -15.28 | 6.5618 | -2.33 | 1.98e-02 |
| Treatment = *Serratia* | -2.48 | 0.8354 | -2.97 | 2.96e-03 |
| Treatment = *Staphylococcus* | -4.36 | 0.9241 | -4.72 | 2.32e-06 |
| Sex = female | 1.10 | 0.0942 | 11.67 | 1.83e-31 |
| Sex = male | 1.55 | 0.1033 | 15.05 | 3.22e-51 |

Scale female=3, male=4.73; Gaussian distribution; Loglik(model)=-293.1; Loglik(intercept only)=-304.3; Chisq=22.4 on 3 degrees of freedom; p=5.4e-05; 4 Newton-Raphson Iterations; n =108 (9 observations deleted due to missingness); *Serratia* n=49; *Staphylococcus* n=30; *Enterococcus* n=29; survreg(formula = ful ~ Pupal.weight + strata(Sex) + Treatment, data = fuldf, dist = "gaussian").
